# Modular Framework for Responsive and Explainable Robotic Assistance with Intention Prediction Using Human-Centric Digital Twins

**DOI:** 10.3390/s26123810

**Published:** 2026-06-15

**Authors:** Usman Asad, Azfar Khalid, Waqas Akbar Lughmani, Shummaila Rasheed, Muhammad Mahabat Khan

**Affiliations:** 1 Department of Mechanical Engineering, Capital University of Science and Technology, Islamabad 45750, Pakistan; shummaila@cust.edu.pk (S.R.); drmahabat@cust.edu.pk (M.M.K.); 2 Digital Innovation Research Group, Department of Engineering, School of Science & Technology, Nottingham Trent University, Nottingham NG11 8NS, UK; azfar.khalid@ntu.ac.uk; 3 Department of Engineering, Birmingham City University, Birmingham B4 7XG, UK; waqas.lughmani@bcu.ac.uk

**Keywords:** human–robot collaboration, proactive assistance, vision-language models, intent prediction, explainable AI, digital twins, industry 5.0

## Abstract

Proactive robotic assistance in human–robot collaboration (HRC) requires systems that can perceive evolving task contexts, anticipate user needs, and intervene appropriately without disrupting human workflow. We present the Agentic Unified Robotic Assistance (AURA) Framework, which couples Large Language Model (LLM) reasoning grounded by Standard Operating Procedures (SOPs) with a modular layer of specialized Intent, Motion, Perception, Sound, Affordance, and Performance Monitors that supply structured context to a central decision-making module, making the framework reconfigurable and auditable without retraining or re-prompting. We introduce a human-in-the-loop teleoperation data collection methodology and an offline evaluation scheme with an Appropriateness Score (A-Score) tailored to proactive intervention timing, and release a benchmark dataset of annotated multimodal HRC episodes containing workspace and robot wrist camera videos, robot joint states, and labeled intervention events. Across three tasks of varying complexity, we observe progressive gains in intent prediction and decision-making as the modules are supplied with richer grounded context (prior-state memory and tracked object locations), with Combined F1 rising by over 20 points between context-poor and context-rich conditions. The structured grounding allows lightweight multimodal backbones such as Gemini 3.1 Flash Lite to perform on par with heavier reasoning-tier models at roughly one-fifth the inference latency. Together, these contributions establish a scalable framework, benchmark, and evaluation methodology for advancing proactive robotic assistance in collaborative environments.

## 1. Introduction

### 1.1. The Shift to Proactive HRC in Industry 5.0

The manufacturing landscape is undergoing a profound transformation, moving beyond the efficiency-driven paradigm of Industry 4.0 towards the human-centric, sustainable, and resilient principles of Industry 5.0 [1]. This evolution places renewed emphasis on the human operator not as a mere component in a mechanized process, but as the central agent whose cognitive skills, dexterity, and adaptability are paramount [2]. In this context, Human–Robot Collaboration (HRC) is evolving from simple coexistence, where robots operate behind safety fences, to more advanced symbiotic and proactive paradigms [1,3]. The goal is no longer simply to automate repetitive tasks. Instead, it is to create dynamic partnerships. These partnerships combine the endurance, precision, and strength of robots with the nuanced reasoning and problem-solving abilities of humans. This synergy is particularly critical in high-mix, low-volume (HMLV) manufacturing environments, where traditional, rigid automation is often impractical and economically unviable [4].

Despite the high pace of technological advancements in AI and Robotics, significant obstacles remain, limiting the adoption of embodied AI by industry. Specifically, there is a critical need to have modular, explainable actions which are not just opaque outputs of a black box. Operators in industrial settings require transparency to trust robotic partners; systems that function as black boxes prevent verification and complicate error recovery. Therefore, the adoption of these technologies in SMEs relies on bridging the gap between high-performance neural models and the requirement for interpretability and safety [5,6].

A key technological enabler for this shift is the concept of the Human-Centric Digital Twin (HCDT). An HCDT is a high-fidelity virtual model encompassing not just the physical environment and robot, but also the human operator, including their actions, intentions, and even cognitive states [2]. By creating a predictive model of the human, an HCDT can serve as the foundation for a robotic system. Such a system does not merely react to commands; it actively anticipates the operator’s needs, paving the way for truly proactive assistance.

### 1.2. From Intent Prediction to Proactive Action

Previous research has made significant strides in the foundational challenge of building such HCDTs. Asad et al. [7] introduced a modular framework capable of forecasting human intent and generating plausible future motion from multimodal inputs, including video streams and structured task knowledge represented as action graphs. This framework demonstrated that by leveraging Vision-Language Models (VLMs) and carefully engineered context, it is possible to achieve robust understanding of an operator’s position within a task sequence and predict their immediate future actions with considerable accuracy.

However, predicting intent is only the first, albeit critical, step. The ultimate promise of an HCDT lies in its ability to drive intelligent action. While prior work established that a system can predict what an operator is likely to do next, it left open the crucial subsequent question: what should a collaborative robot do with that prediction? The next frontier in HRC research, and the focus of this work, is to bridge the gap between passive prediction and active, intelligent intervention. This involves developing a framework that can translate predicted intent into timely, appropriate, and unsolicited robotic assistance, thereby transforming the robot from a reactive tool into a proactive partner.

### 1.3. Evaluation in Proactive HRC

As the field pushes towards more sophisticated proactive behaviors, a significant methodological gap has emerged: the lack of adequate evaluation metrics. Conventional metrics for HRC, while valuable, are largely rooted in a reactive or command-driven paradigm. Measures such as task completion time, human or robot idle time, and error rates are effective at quantifying the efficiency of a collaborative process [8]. Foundational work on teamwork fluency [9] broadened this view by proposing complementary measures of joint-action quality, namely the percentage of human–robot concurrent motion, human idle time, and the functional delay between a human action and the robot’s consequent action. However, they are fundamentally insufficient for capturing the quality or appropriateness of proactive, unsolicited assistance.

A robot could, for example, aggressively take over the majority of an operator’s tasks, resulting in optimized completion times and minimal human idle time. While conventional metrics would classify such a system as highly successful, from a human-centric perspective, this interaction represents a failure if the aggressive takeover results in actions that do not align with the human’s specific requirements or preferences. In these instances, the robot becomes intrusive and disempowering, ultimately undermining the collaborative synergy it is intended to support. This highlights a central challenge in the field: existing metrics often fail to distinguish between genuinely helpful assistance and well-timed but unwelcome interference, as they do not account for whether the intervention was truly desired or delivered in a manner consistent with the operator’s intent [10,11].

### 1.4. Benchmark Data for Proactive HRC

Beyond measurement challenges, a significant constraint in proactive Human–Robot Collaboration (HRC) research is the lack of standardized benchmark datasets. While fields such as computer vision and natural language processing utilize established datasets to facilitate reproducible comparative studies, proactive robotics lacks equivalent resources. For instance, while systems like MOSAIC [12] demonstrate effectiveness in assistive tasks, they are typically presented as standalone system evaluations rather than as publicly available, annotated datasets with ground-truth intervention timings. Furthermore, most existing HRC datasets focus on reactive interactions, teleoperation, or kinesthetic teaching, which do not fully capture the anticipatory decision-making required for proactive assistance. Addressing this issue requires both new evaluation metrics and new methods for collecting ground-truth data, which accurately represent the appropriate context-dependent proactive behavior.

### 1.5. Contributions

This paper makes the following contributions. First, we present the Agentic Unified Robotic Assistance (AURA) Framework, a comprehensive agentic architecture for proactive robotic assistance that integrates real-time multimodal perception with LLM-based reasoning, grounded by Standard Operating Procedures (SOPs) as domain-specific constraints. Second, we introduce a Monitor-Based Architecture, a modular system comprizing specialized monitors (Intent, Motion, Perception, Sound, Affordance, Performance) that continuously inform a central decision-making module, enabling explainable and context-aware decisions. Third, we define the Appropriateness Score (A-Score), a metric that grades each intervention by how well its timing and action match the ground truth and penalizes predictions made during expected wait periods, validated against common alternatives on a synthetic adversary suite. Fourth, we introduce a human-in-the-loop teleoperation data collection methodology for creating ground-truth recordings of proactive assistance, where a human operator controls the robot to help a task performer, capturing expert judgment on intervention timing and action selection. Fifth, we release a set of annotated evaluation recordings for proactive HRC, comprising synchronized multimodal episodes (workspace video, robot wrist camera, robot joint states) with annotated intervention events, enabling reproducible evaluation of proactive assistance algorithms. Finally, we demonstrate an Offline Evaluation Pipeline that processes recorded data through the framework and compares predicted interventions against human operator ground truth, providing quantitative metrics for system performance.

## 2. Related Work

### 2.1. Evolution of Collaborative Paradigms

Human–Robot Collaboration has progressed through several distinct stages, each characterized by increasing integration and intelligence. The earliest stage was coexistence, where industrial robots operated in isolation, separated from humans by physical barriers for safety [3]. The advent of collaborative robots (cobots) enabled sequential collaboration, where humans and robots could share a workspace but worked on a task in turn, and simultaneous collaboration, where they could work on the same product at the same time but on different sub-tasks [13].

More recently, the field has moved towards advanced paradigms. Symbiotic HRC describes systems where humans and robots engage in tightly coupled interactions, often involving active collision avoidance and adaptive control based on multimodal communication (e.g., voice, gesture) [1,3]. This paradigm, however, often maintains a master/slave dynamic, with the robot reacting to human commands or a predefined plan. The ultimate goal, and the focus of this research, is Proactive HRC. This paradigm is defined by anticipatory, self-initiated, and change-oriented robot behavior [14]. In a proactive system, the robot does not wait for an explicit request; it anticipates the user’s needs, goals, and potential difficulties, and offers unsolicited assistance to achieve a shared objective [1,15].

### 2.2. Architectures for Intent-Driven HRC

The development of proactive systems relies on sophisticated software architectures capable of interpreting human intent and planning synchronized actions. The Proactive Assistance through Action-Completion Estimation (PACE) framework by De Lazzari et al. [16] combines Dynamic Time Warping (DTW) with correlation analysis on hand trajectories to estimate human task progress, and trains a reinforcement learning policy from limited demonstrations to align robot actions with that progress. This system estimates the completion time of human actions in real time to minimize robotic idle time and improve interaction fluency. David et al. [4] presented the Collaborative Agents for Manufacturing Ontology (CAMO), which uses description-logic semantic modeling and a multi-agent system to enable self-organization in human–robot teams via consensus-driven role negotiation over agent capabilities. Their architecture incorporates a projector-based mixed-reality interface to project robot intentions as visual cues on the workpiece. Umbrico et al. [8] developed a framework that transitions from general human-aware control to personalized user-aware control. Their approach combines an ontology-based user model with AI task and motion planning to adapt robot behavior to the skills and preferences of individual operators. Across these works, proactive coordination is achieved through hand-engineered representations such as RL policies, ontologies, and user models.

### 2.3. The Grounding Problem: Connecting Language to Action

Large Language Models (LLMs) and Vision-Language Models (VLMs) are trained on vast datasets from the internet, endowing them with remarkable semantic knowledge and reasoning capabilities. However, this knowledge is disembodied; the models lack direct experience with the physical world, its constraints, and the consequences of actions [17]. An LLM might plausibly suggest “using a vacuum cleaner” to clean a liquid spill, an action that is nonsensical for a typical manipulator arm [18].

The SayCan framework was a seminal contribution to solving this problem [19]. It combines the strengths of LLMs and robotic learning: for a given high-level instruction, the LLM provides a probability distribution over available robot skills indicating semantic relevance. Simultaneously, pre-trained value functions provide the probability that each skill can be successfully executed from the robot’s current state. By multiplying these probabilities, SayCan selects an action that is both semantically plausible and physically possible.

Building upon semantic grounding, frameworks like ProgPrompt [20] advance task planning by representing plans as executable Pythonic programs. Unlike the flat list of skills in SayCan, ProgPrompt utilizes the structured reasoning of LLMs to generate loops, assertions, and error-recovery steps, grounding these programmatic plans in the specific capabilities and objects available within the robot’s environment.

The advent of powerful VLMs represents a further step, as they can directly process visual input alongside text, allowing them to reason about objects and scenes in front of the robot. This reduces the need for separate, pre-trained value functions, as the model can implicitly learn affordances from visual data. Techniques like affordance prompting have been proposed to explicitly encourage the LLM to reason about physical consequences of planned actions [21].

### 2.4. Vision-Language-Action Models for Robot Control

The Vision-Language-Action (VLA) paradigm represents a fundamental departure from classical robotics, aiming to unify visual perception, natural language understanding, and embodied control within a single, end-to-end learning framework [22]. Models like RT-2 demonstrated that by representing robot actions as text tokens, a VLM can directly leverage its semantic understanding for control, exhibiting emergent capabilities such as understanding symbols and spatial relationships [23].

Recent developments include diffusion policies [24], which model robot action sequences as a conditional denoising process, and large-scale generalist policies such as $\pi_{0}$ [25] and OpenVLA [26], which fine-tune vision-language backbones on cross-embodiment manipulation data.

### 2.5. Datasets and Benchmarks for HRC

The robotics community has developed numerous datasets for manipulation, navigation, and human activity recognition, yet datasets specifically designed for evaluating proactive human–robot collaboration remain scarce. Existing HRC datasets primarily address reactive scenarios: teleoperation datasets capture human demonstrations for imitation learning, kinesthetic teaching datasets record physical guidance for skill transfer, and activity recognition datasets label human actions without considering robotic intervention [27].

Recent systems like MOSAIC [12] demonstrate proactive assistance in cooking scenarios, with the robot anticipating user needs and offering help. However, such work evaluates performance through end-to-end task success rates rather than releasing annotated datasets with intervention timings that would enable comparison of the underlying decision algorithms. Similarly, PACE [16] evaluates synchronization quality but does not provide datasets for reproducing or benchmarking against their results.

The challenge in creating proactive HRC datasets lies in defining ground truth. Unlike object detection or action recognition, where annotations are relatively objective, determining the optimal moment and modality for proactive assistance involves subjective human judgment that depends on task context, operator skill level, and individual preferences. This paper addresses this challenge through a human-in-the-loop teleoperation methodology that captures expert human decisions as ground truth, enabling quantitative evaluation of proactive assistance algorithms.

The existing literature also lacks publicly available ground-truth data for proactive interventions. To address this, we release a set of annotated evaluation recordings alongside our framework. Captured via a human-in-the-loop teleoperation methodology, these multimodal recordings contain roughly 30 min of high-density execution data across two distinct composite layup tasks. The recordings synchronize 125 Hz robot joint states, 360-degree wrist camera footage, and third-person workspace video, and include continuous task-state annotations and precise temporal timestamps for robot interventions.

As summarized in Table 1, while previous architectures have successfully tackled individual components of Human-Centric Digital Twins, AURA is uniquely positioned at the intersection of proactive behavior, vision-language reasoning, and procedural adherence. While frameworks like SayCan and ProgPrompt demonstrate the reasoning power of LLMs for deconstructing tasks, they primarily focus on unconstrained generative planning where the robot imagines a sequence of steps based on commonsense knowledge. Conversely, systems like PACE and CAMO demonstrate proactive synchronization but rely on limited demonstration-trained models or rigid, hand-engineered ontologies rather than the open-vocabulary reasoning afforded by modern VLMs. Furthermore, while MOSAIC introduces VLM-driven proactive assistance, it primarily focuses on open-ended domestic tasks, such as cooking, that lack the strict sequential and safety requirements of formal Standard Operating Procedures (SOPs).

More recent proactive systems converge on language-model reasoning but follow different architectural routes. LIT [28] tracks human intent with a language-model-driven Bayesian filter, allowing a kitchen robot to anticipate the next step. AssistantX [29] coordinates several language-model agents that plan, act, and reflect in order to proactively seek human help in an office setting. NIABench [30] frames assistance as a joint timing-and-action decision and trains a ranking model to choose well-timed, non-intrusive actions. RoboOmni [31] instead takes an end-to-end route, training a single model on a large corpus to infer intent from speech, ambient sound, and vision.

**Table 1:** Qualitative comparison of AURA with state-of-the-art HRC frameworks.

Framework	Architecture Style	Proactive Assistance	VLM/LLM-Driven	SOP-Grounded	Pre-Training Required	Reconfigurable	Explainable	Upgradable	Open Dataset
SayCan [19]	Modular (LLM + RL)	No	Yes	No	Yes (RL)	No	No	Yes	No
CAMO [4]	Multi-Agent (Ontology)	Yes	No	Yes	No (rules)	No	Yes	No	No
MOSAIC [12]	Modular (LLM + Vision)	Yes	Yes	No	No (Zero-shot)	No	No	Yes	No
LIT [28]	Modular (Bayesian Filter)	Yes	Yes	No	No (Zero-shot)	Yes	No	Yes	No
AssistantX [29]	Multi-Agent (LLM)	Yes	Yes	No	No (Zero-shot)	No	Yes	Yes	Yes
PACE [16]	Modular (DTW + RL)	Yes	No	No	Yes (RL)	No	No	No	No
RoboOmni [31]	End-to-End (VLA)	Yes	Yes	No	Yes (corpus)	No	No	No	Yes
NIABench [30]	Modular (LLM + Ranker)	Yes	Yes	No	Yes (ranker)	No	No	No	Yes
AURA (Ours)	Modular	Yes	Yes	Yes	No (In-context)	Yes	Yes	Yes	Yes

There remains a notable absence of generalized frameworks capable of bridging the flexible reasoning of VLMs with the explicit rules of structured procedural tasks. AURA addresses this gap by introducing a modular, highly extensible architecture. By utilizing a neuro-symbolic approach where the VLM acts as a reasoner constrained by a symbolic Directed Acyclic Graph (DAG) derived from task SOPs, AURA ensures both proactive assistance and procedural compliance. Crucially, this modular design allows the framework to be easily adapted to entirely new procedural workflows without structural changes, relying instead on interchangeable task configurations and specialized monitors. Two further properties distinguish AURA from these systems. Whereas SayCan needs pre-trained affordance value functions, PACE a policy learned from demonstrations, RoboOmni a large training corpus, and NIABench a trained ranker, foundation-model systems such as MOSAIC, LIT, AssistantX, and AURA require no task-specific pre-training. Moreover, because the architecture is a modular composition of monitors, newer vision-language, segmentation, or pose-estimation models can be substituted as they emerge, whereas a monolithic end-to-end system such as RoboOmni must be retrained to benefit from a better component.

## 3. Framework Architecture

This section details the theoretical and structural foundations of the Agentic Unified Robotic Assistance (AURA) framework. We first formalize the problem of proactive robotic assistance and establish our evaluation metric before providing a comprehensive overview of the system’s modular layers.

### 3.1. Problem Formulation

#### 3.1.1. Need-Based Proactive Assistance

We formalize proactive robotic assistance as follows. A human operator and a collaborative robot share a workspace containing task-relevant objects. Their joint activity is governed by a Standard Operating Procedure (SOP). This is initialized by specifying a structured task configuration: a Directed Acyclic Graph (DAG), a state schema tracking task progress, a catalog of robot skills with preconditions and effects, and an initial scene description. Section 3.7 details this configuration.

At each timestep *t*, the system observes

(1) $$x_{t}=\left\{I_{t},p_{t}^{H},u_{t},s_{t}\right\},$$

where $I_{t}$ is the visual frame, $p_{t}^{H}$ the human pose, $u_{t}$ any audio (utterance) input, and $s_{t}$ the current task state. The framework implements a decision policy π that selects actions $a_{t}∼\pi \left(x_{t}\right)$ to maximize the appropriateness of proactive interventions over the task duration:

(2) $$\max\sum_{t\in T}A_{score}\left(a_{t},x_{t}\right),$$

where T={1,2,…,T} is the set of all decision time steps from task onset (t=1) to task completion (t=T).

#### 3.1.2. Decision Types

The robot must decide among several action types at each decision point: WAIT (continue observing without acting), EXECUTE_ACTION (perform a physical manipulation task), ASK_HUMAN (request clarification when uncertain), SPEAK (communicate information proactively), COMPLETE_TASK (mark a task node as finished), or ABORT (halt current action for safety).

#### 3.1.3. The Appropriateness Score (A-Score)

In an offline evaluation setting, the framework operates open-loop with respect to the robot’s physical execution state: the decision engine emits a predicted action on every decision tick and continues to do so until the recorded visual state shows the action has been completed, at which point the action is no longer deemed necessary. Interventions are therefore treated as continuous time intervals rather than discrete trigger events. Consecutive ticks that emit the same action identifier are merged into a single contiguous prediction interval.

Let $G={\left\{\left(a_{i}^{*},I_{i}^{*}\right)\right\}}_{i=1}^{N_{gt}}$ denote the set of ground-truth interventions, where each $I_{i}^{*}=\left[t_{s,i}^{*},t_{e,i}^{*}\right]$ is the expert intervention window of length $L_{i}=t_{e,i}^{*}-t_{s,i}^{*}$ and $a_{i}^{*}$ the expert skill. Let $P={\left\{\left({\hat{a}}_{j},{\hat{I}}_{j}\right)\right\}}_{j=1}^{N_{pred}}$ denote the set of contiguous prediction intervals produced by the agent over a recording of total duration *T*, and let $T_{wait}=T-\sum_{i}L_{i}$ be the implicit WAIT time.

The score partitions the timeline into one slice per ground-truth event plus a single WAIT slice and grades each slice on its own quality factor. With effective normalizer $Z=\sum_{i}L_{i}+w_{w}T_{wait}$, the Appropriateness Score is

(3) $$A_{score}\;=\;\underset{\mathrm{task}\;\mathrm{term}}{\underset{︸}{\sum_{i=1}^{N_{gt}}\frac{L_{i}}{Z}\;q_{i}}}\;+\;\underset{\mathrm{wait}\;\mathrm{term}}{\underset{︸}{w_{w}\;\frac{T_{wait}}{Z}\;q_{wait}}}.$$

**Per-event quality** $q_{i}$**.** For each GT event, we pick the same-skill prediction whose start- and end-time errors give the highest Gaussian decay,

(4) $$q_{i}\;=\;\max_{{\hat{I}}_{j}\;:\;{\hat{a}}_{j}=a_{i}^{*}}\exp\;\left(-\left({\left(\Delta s_{j}/\sigma_{i}\right)}^{2}+{\left(\Delta e_{j}/\sigma_{i}\right)}^{2}\right)\right),\;\sigma_{i}=\alpha \;L_{i},$$

where $\Delta s_{j}=\left|{\hat{t}}_{s,j}-t_{s,i}^{*}\right|$ and $\Delta e_{j}=\left|{\hat{t}}_{e,j}-t_{e,i}^{*}\right|$. Tying the timing scale to $L_{i}$ makes the tolerance proportional to the event’s own duration. If no prediction shares the GT skill, $q_{i}=0$. Multiple GT events of the same skill may claim the same prediction, but the timing decay prevents one well-placed prediction from earning credit for a far-away GT event.

**Wait quality** $q_{wait}$**.** Let $\Phi_{a}$ denote the union of GT intervals with skill *a*. The pollution time of a prediction ${\hat{I}}_{j}$ is its length minus its overlap with $\Phi_{{\hat{a}}_{j}}$:

(5) $$T_{fp}\;=\;\sum_{j}\left(|{\hat{I}}_{j}\left|-\right|{\hat{I}}_{j}\cap \Phi_{{\hat{a}}_{j}}|\right).$$

A prediction whose skill matches a GT event but whose timing is off contributes a small $q_{i}$ and its non-overlapping mass becomes pollution; a prediction whose skill is absent from G is pure pollution. The wait term decays smoothly with $T_{fp}$,

(6) $$q_{wait}\;=\;\exp\;\left(-T_{fp}/\left(\beta \;T_{wait}\right)\right),$$

and we set $q_{wait}≜1$ when $T_{wait}=0$.

The score is therefore tolerance-free (no hard match cliff), duration-weighted (missing a long event costs more than missing a short one), and wait-aware (the wait term has its own quality factor). The effect of parameters such as α, β, and $w_{w}$, and a comparison against alternative metrics, is presented in Section 5.1.

### 3.2. System Overview

AURA (Agentic Unified Robotic Assistance) is a modular, event-driven architecture centered on a decision-making brain coordinating specialized monitors. Its design follows three principles: separation of concerns between perception, reasoning, and action; explainability through inspectable decision rationale; and SOP grounding, where domain knowledge constrains LLM reasoning.

**Figure 1:**
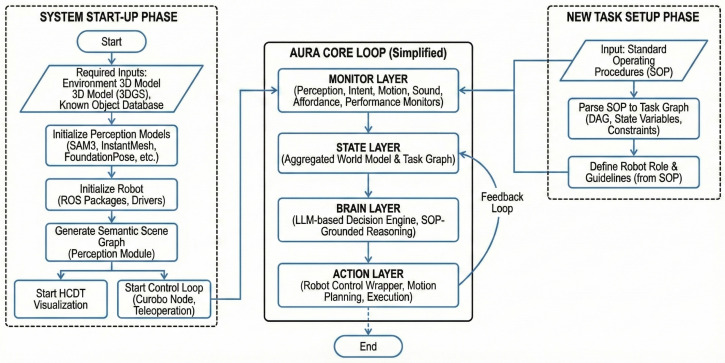
System initialization and task setup workflow.

Initialization (Figure 1) proceeds in two parallel phases that converge to activate the runtime loop: the task configuration (Section 3.7) is loaded, and the Human-Centric Digital Twin (HCDT) is instantiated with the robot model and known objects of interest, optionally augmented by a 3D Gaussian Splatting environment model (Section 4.2).

**Figure 2:**
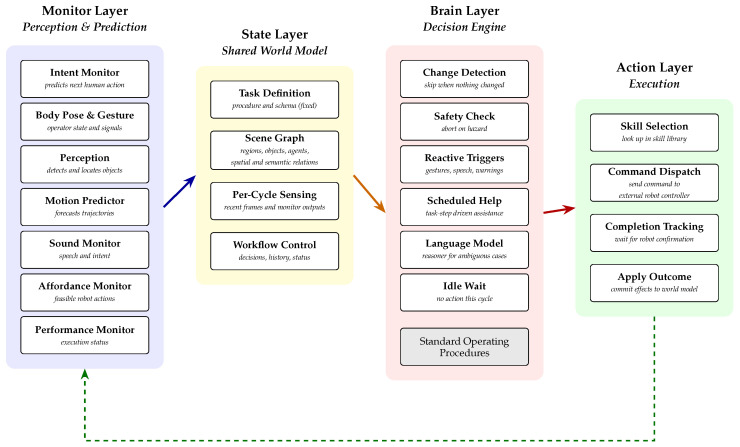
High-level architecture of the AURA framework.

Figure 2 illustrates the architecture: a Monitor Layer (perception and prediction), State Layer (shared world model), Brain Layer (decision engine), and Action Layer (motion planning and execution).

### 3.3. Monitor Layer

The Monitor Layer is an open-ended set of modules that process multimodal inputs and write evidence into the shared state. The active set is selected per deployment via the task’s workflow_config (Section 3.7). The modules below are those used in the experiments reported here.

#### 3.3.1. Intent Monitor

**Figure 3:**
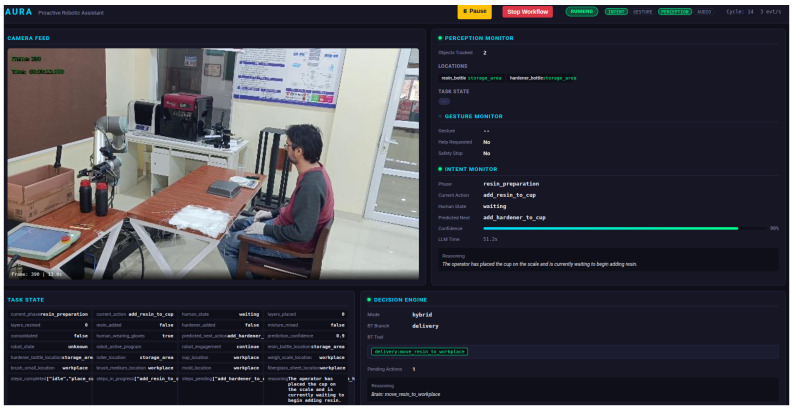
Example of intention monitoring dashboard output for a task involving manual layup in composite manufacturing.

The Intent Monitor queries a VLM over a rolling window of recent frames, the task DAG and state schema, and the previous cycle’s output. It returns a JSON object that classifies every DAG step as completed, in-progress, or pending, names the predicted next action, sets the task-specific state variables, and gives a brief reasoning trace (see Figure 3). An optional workspace anchor image grounds object identities and spatial layout.

#### 3.3.2. Perception Module

The generic Perception Module wraps SAM 3 [32] for text-prompted segmentation and optionally invokes a VLM for scene-level description, returning bounding boxes, masks, confidences, and labels. These generic outputs lack task-level semantics: a detector prompted with “bottle” cannot distinguish visually identical instances, nor can it infer which table serves as storage versus workspace.

**Figure 4:**
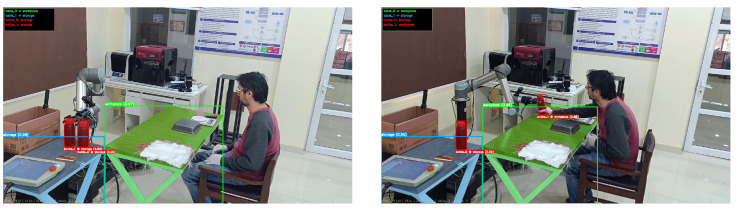
Task-specific perception output for the hand layup task. (**Left**): both bottles detected on the storage table. (**Right**): following a transfer, one bottle is re-assigned to the workplace. Table regions are shown as colored mask overlays (blue: storage, green: workplace); bottles are marked in red with their assigned region.

The framework therefore supports task-specific perception monitors that compose the generic module with spatial heuristics. The hand layup monitor (Section 4.3.1), for example, uses only “table” and “bottle” prompts and resolves identity geometrically (see Figure 4): on the first frame, the table containing the most bottle centroids is labelled “storage”; in subsequent frames, tables are matched to their known regions by nearest-neighbor assignment. Bottle-to-table assignment uses the mask overlap ratio [33]

(7) $$\rho \left(B_{i},T_{j}\right)=\frac{|M_{B_{i}}\cap M_{T_{j}}|}{|M_{B_{i}}|},$$

with fallbacks to centroid containment and Euclidean proximity, and the most recent definitive assignment is retained when no heuristic matches.

**Figure 5:**
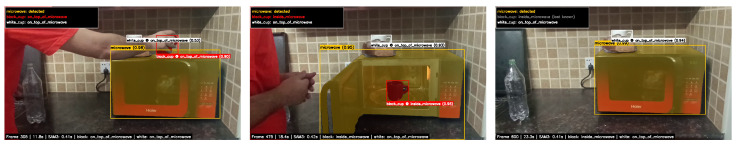
Perception under occlusion in the microwave task. (**Left**): the white cup, partially occluded on top of the microwave. (**Middle**): the black cup inside the open microwave. (**Right**): door closed and appliance running, with the black cup fully occluded and yielding no mask.

**Figure 6:**
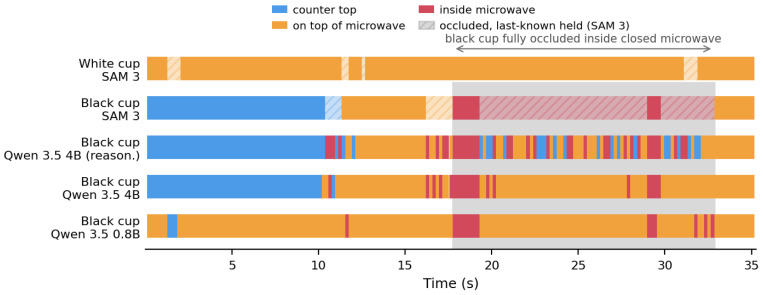
Comparison of estimated cup locations over time.

The last-known-state retention rule above is what keeps the perception layer usable under partial and full occlusion. In a microwave-heating scenario (Figure 5), a white cup that is only partially visible is still segmented and tracked, and a black cup is resolved by the overlap ratio of Equation (7) while it remains visible. Once the door closes and the cup is fully occluded, no mask is produced, and the monitor holds its last inside-the-microwave label rather than hallucinating the cup elsewhere, giving downstream policies a stable location for as long as the object does not move. A per-frame VLM baseline cannot represent this persistence. Across the 66 fully occluded frames, only the 4B reasoning model recovers the correct location (inside the microwave), and only in 26% of frames, with  a latency of 7 s per frame; the lighter and non-reasoning variants stay wrong throughout the occlusion duration (Figure 6). The monitor instead tracks the cup at 0.42 s per frame, and its decisions remain auditable, tracing to a mask, an overlap value, and an explicit last-known-location rule.

#### 3.3.3. Zero-Shot 6-DOF Pose Estimation

**Figure 7:**
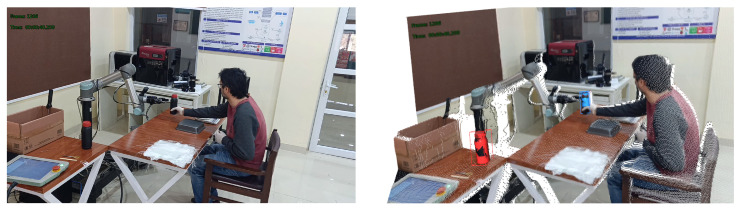
Zero-shot 6-DOF pose estimation from monocular RGB video. (**Left**): input frame. (**Right**): estimated poses rendered as textured mesh overlays on the scene point cloud, combining Any6D [34], Depth Anything 3 [35], SAM 3 [32], and a SAM 3D [36] reconstructed mesh.

For 6-DOF pose estimation of novel objects, the Perception Module composes Any6D [34] (model-free pose registration with iterative refinement and a learned scoring network), SAM 3 [32] (text-prompted per-frame masks), Depth Anything 3 [35] (monocular metric depth), and SAM 3D [36] (mesh reconstruction from a reference image) into a single pipeline that operates on raw monocular RGB video without an instance mesh, sensor depth, or calibrated intrinsics. The reconstructed mesh is auto-scaled to metric units via an isotropic procedure that compares oriented-bounding-box extents of the mesh against the depth-lifted scene cloud, followed by a multi-scale search refined by Any6D’s scoring network so that the known geometry is never deformed by anisotropic stretching. Per-frame registration is run independently on each frame for which a SAM 3 mask is available, with Any6D’s temporal tracking used only as a fallback. Estimated poses are rendered as textured mesh overlays via differentiable rasterization [37] for visual verification (Figure 7). In the framework, this pipeline is primarily used offline to initialize the digital-twin scene from a reference video; using it for real-time perception inside the decision loop is possible in principle but compute-bound on current hardware. Empirically, monocular metric depth is the limiting factor for fully RGB-only operation: replacing the ground-truth mesh with a SAM 3D reconstruction or the calibrated intrinsics with DA3 predictions introduces only minor degradation, but when DA3’s predicted focal length disagrees with the true intrinsics, the resulting depth-scale error propagates directly into pose error.

#### 3.3.4. Sound Monitor

The Sound Monitor provides a bidirectional conversational interface. It runs as a long-lived background process maintaining a streaming session with a live audio LLM, decoupled from the perception–decision loop so that speech latency cannot stall it. Bound to the task-specific system instruction and the robot’s skill catalog, it maps spoken commands (e.g., “bring me the resin bottle”) directly to skill invocations through the same control interface used by the decision engine. Contextual observations from the human (e.g., “the hardener is already on the bench”) are registered as updates to the shared world model. Transcribed utterances and world-model updates are merged into the Semantic Scene Graph once per cycle, making speech a first-class input to reasoning.

#### 3.3.5. Body Pose, Activity, and Gesture Monitors

The Body Pose Monitor produces per-frame human poses used for two downstream functions: a lightweight activity gate on the expensive intent call, and a task-dependent safety check that can interrupt robot motion. Whole-body pose inference is delegated to an external Fast-SAM-3D-Body [38] service over a local channel, isolating its dependencies and allowing graceful degradation: when the service is unavailable, the activity gate falls back to always-on so the intent pipeline does not stall.

Activity Detection gates the intent call on a Boolean activity signal. The current implementation thresholds the mean Euclidean displacement of the operator’s 2D pose keypoints across consecutive frames against a configurable pixel value; any classifier consuming the pose stream and emitting the same gate signal can be substituted.

The Gesture Monitor uses MediaPipe Gesture Recognition to detect hand gestures (e.g.,  Open_Palm, Pointing_Up, Thumb_Up, Victory) from the image stream. In our implementation, an optional person detector crops to the operator before recognition; Open_Palm and Pointing_Up are used as safety-stop signals, while Thumb_Up and Victory signal resume.

#### 3.3.6. Affordance and Performance Monitors

The Affordance Monitor assesses which actions are currently feasible given the robot’s configuration, object states, and environmental constraints, producing a filtered set of motion primitives and learned skills available to the decision engine. The Performance Monitor tracks action execution progress, detects discrepancies between expected and actual outcomes, flags stalled actions via timeouts, and classifies failures.

### 3.4. State Layer

**Figure 8:**
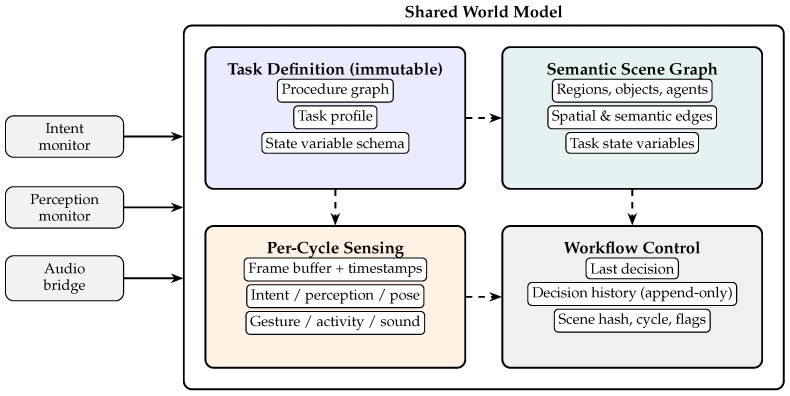
The shared world model passed through each cycle of the workflow.

The State Layer maintains a single shared world model read by all monitors and the decision engine, passed from one stage of the cycle to the next. It is organized into four parts (Figure 8): (i) the task definition (procedural graph, task profile, state-variable schema) loaded at start-up; (ii) the Semantic Scene Graph, the single source of truth for spatial and relational reasoning, containing workspace regions, objects, agents, their spatial and semantic relations, and a flat dictionary of task state variables; (iii) per-cycle sensing, a rolling buffer of recent frames with the latest result from each active monitor; and (iv) workflow control, comprising the most recent decision, an append-only decision history, a hash of the previous scene graph used to skip redundant reasoner calls, the cycle counter, and completion or error flags. Closed-loop LLM planners such as Inner Monologue [39] carry similar memory as natural-language feedback appended to each prompt; here, it is held as typed fields in the SSG.

The Semantic Scene Graph is refreshed each cycle from the active monitors (e.g., the intent and perception monitors) and external bridges (e.g., the audio bridge). Since these run in parallel (Section 3.5), each shared field has a reducer that can append, perform a key-wise merge, or keep the latest write. This avoids concurrent-write conflicts and preserves a complete decision log.

### 3.5. Brain Layer: Decision Engine

The runtime is a LangGraph state machine executing a continuous *sense–decide–act* loop over the shared world model (Section 3.4).

#### 3.5.1. Workflow Topology

Each cycle begins by capturing the next frame into a rolling buffer, then fans out into the active sensing branches in parallel. Slow monitors are gated by lightweight signals and may be dispatched to background workers so they do not stall the loop.

The branches converge at an **Update SSG** node that fuses outputs into the Semantic Scene Graph: predicted state variables and DAG progress from the Intent Monitor, vision-grounded locations from the perception monitor, and pending events drained from the audio bridge. A **Change Detection** step then hashes a compact digest of locations, robot status, completed steps, and intent phase, invoking the decision engine only when the hash changes. When an action is produced, it is dispatched by an **Execute Action** node that looks up the corresponding program, marks the robot busy, issues the control request, and defers post-conditions until the robot status poll confirms physical completion. A final node checks whether a terminal DAG node, error, or cycle limit has been reached, and otherwise loops back to capture.

#### 3.5.2. Hybrid Behavior Tree and LLM Decision Engine

Routing every decision through a VLM is expensive, slow, and non-deterministic in routine cases. The decision engine is therefore a hybrid behavior tree, compiled automatically from the task configuration as a priority selector over five branches: (1) safety, with one leaf per declared safety rule that aborts the current action when its hazardous condition is met; (2) reactive events that are time or event-triggered (timers, help gestures, utterances); (3) automatic skill dispatch, with one leaf per robot skill that runs the skill directly, without calling the LLM, when its declared trigger steps and SSG preconditions are satisfied and the robot is idle; if more than one skill is eligible at the same tick, the conflict is handed to the LLM; (4) LLM fallback, invoked only when the situation is ambiguous, that is, on unresolved conflicts, unhandled gestures or utterances, or low intent confidence; and (5) a guaranteed idle wait.

Three operating modes are supported: hybrid (default), BT-only (the LLM fallback is replaced by a clarifying-question leaf, yielding a fully deterministic policy), and LLM-only. All modes return the same structured output: an action identifier, optional target, confidence, and reasoning trace, keeping logging and evaluation unchanged. The reasoning trace lists the branches traversed, making every action explainable and auditable.

#### 3.5.3. Decision Principles

The engine is governed by the same principles in both branches: Safety First (safety rules evaluated before any other branch), Human Priority (do not interfere while the human is actively working unless asked), Proactive Help (assist when useful or when the human appears stuck), Communication (ask rather than guess), and Efficiency (respect DAG dependencies, avoid redundant actions).

### 3.6. Action Layer

The Action Layer translates decisions into robot motion through a small set of primitives: MOVE _TO_POSE, EXECUTE_PROGRAM, OPEN_GRIPPER/CLOSE_GRIPPER, and WAIT. Collision-free planning uses cuRobo [40]; the underlying control infrastructure is described in Section 4.1.

Each primitive or stored program is addressable by an identifier and parameter dictionary. This abstraction is deliberately VLA-ready: an EXECUTE_PROGRAM entry can be transparently replaced by a natural-language prompt dispatched to a learned Vision-Language-Action policy, with no change to the upstream decision engine. The framework can therefore mix deterministic scripted behaviors for safety-critical actions with learned policies for dexterous sub-tasks without architectural change.

### 3.7. Task Configuration Schema

Each task is fully specified by a set of JSON files in a dedicated directory, with no code changes required. The schema comprises five files: **task DAG**, encoding the SOP as nodes (identifier, description, dependencies) traversed at runtime to track step availability and completion; **task profile**, mapping abstract actions to robot program files, declaring safety rules, and selecting which monitors are active via workflow_config; **state schema**, defining task-specific state variables (phase, action, progress counters, material flags, robot status) tracked by the Intent Monitor; **robot skills**, a catalog with each skill’s identifier, category, API call, preconditions, effects, estimated duration, and interruptibility, used by the decision engine to filter feasible actions and update the SSG after execution; and **initial scene**, bootstrapping the SSG with workspace regions, objects with initial locations, and agents. A concrete instantiation for composite hand layup is presented in Section 4.3.1.

## 4. Implementation and Experimental Setup

The framework is implemented in Python with LangGraph for state-machine orchestration, ROS 2 Humble as the robot middleware, and Isaac Sim 5.0 for simulation. A single session launcher coordinates the UR5 driver, gripper adapter, motion planner, program executor, REST API, and graphical interface, allowing any subset of capabilities to be activated depending on whether the system runs in autonomous or human-in-the-loop teleoperation mode.

### 4.1. Robot Control and Task Programming Infrastructure

**Figure 9:**
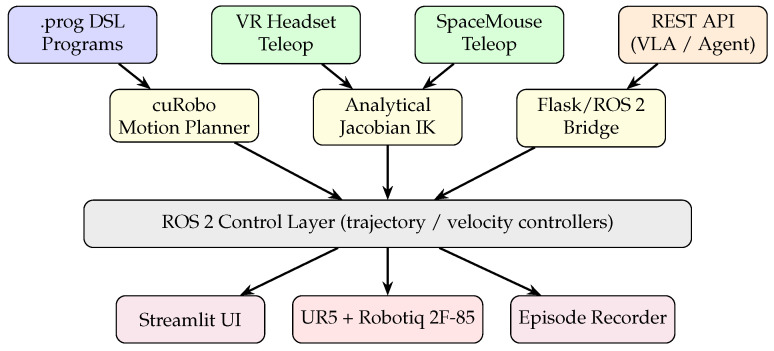
Multi-modal robot control architecture.

Both proactive assistance and ground-truth data collection require a multi-modal control infrastructure that supports rapid authoring of assistance actions, intuitive manual control, and an external interface for autonomous agents. We address this with four input modalities, a domain-specific programming language, VR teleoperation, desktop SpaceMouse control, and a REST API, all converging on a unified ROS 2 control layer (Figure 9).

#### 4.1.1. Domain-Specific Language for Robot Programs

To enable rapid authoring of repeatable assistance actions, we designed a lightweight domain-specific language (DSL) stored as plain-text .prog files. Programs can be written by operators, researchers, or LLMs. The instruction set encompasses commands for moving to Cartesian or joint poses, as well as moving relative to a known reference by a specified direction and distance for predictable approach and retract motions. It includes primitives for gripper actuation, proportional closure, timing control, and speed setting. System-level force control allows compliant-mode activation and deactivation, with motion commands automatically suspending and restoring the compliant state dynamically. The language also provides conditional branching based on gripper state or proximity to known configurations, and supports parameterized sub-programs via template tokens for flexible execution.

**Figure 10:**
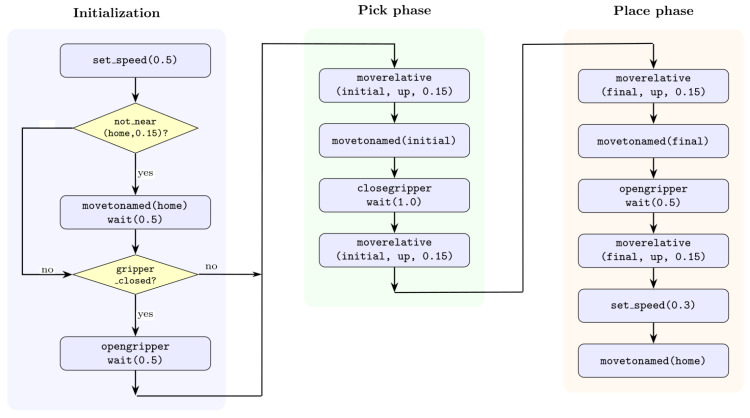
Example generic parameterized robot program for material handling.

A named-positions configuration file stores reusable joint or Cartesian poses. New entries can be appended at runtime; for example, a button on the teleoperation controller records the current configuration, enabling teach-by-demonstration. Figure 10 shows a generic, parameterized object pick-and-place program, combining argument templates, conditional state checks, named positions, and relative motions.

A dedicated program executor parses and step-executes these programs, embedding a cuRobo [40] planner for GPU-accelerated, collision-aware trajectory generation, and exposing ROS 2 services for interactive use and autonomous triggering.

A subtlety in moverelative arises when the offset is computed from a named reference: because the UR5 admits multiple IK solutions for a given pose, a naïve solver may return a joint configuration far from the reference, producing large unexpected motions when the robot subsequently visits the reference itself. The executor therefore seeds the IK solver with the reference configuration $q_{ref}$ and applies a regularized IK

(8) $$q_{target}=\arg\min_{q}{∥\mathrm{FK}\left(q\right)-\left(p_{ref}+d\hat{v},\;r_{ref}\right)∥}^{2}+\lambda {∥q-q_{ref}∥}^{2},$$

biasing the solution toward the same kinematic branch as the reference and yielding short, predictable joint-space trajectories.

The DSL’s simplicity makes it amenable to generation by LLMs: given a task description and the list of named positions, an LLM can produce valid .prog files directly, enabling both rapid prototyping and runtime synthesis of novel action sequences. Code as Policies [41] similarly has the LLM emit control code, but as free-form Python over robot APIs; restricting generation to this small instruction set keeps each program inspectable for the offline review required by industrial SOPs.

#### 4.1.2. Immersive and Desktop Teleoperation

Two complementary teleoperation modalities support human-in-the-loop data collection. A consumer VR headset streams 6-DOF controller poses at 50 Hz; a DROID-style [42] proportional controller maps Cartesian velocity errors to joint velocities via an analytical geometric Jacobian with damped least-squares pseudo-inverse (λ=0.05) for stability near singularities, with a grip-button deadman, trigger-based proportional gripper control, and buttons to save named positions. A desktop 6-DOF input device (SpaceMouse) reuses the same Jacobian-IK pipeline. Both modes bypass trajectory planning and publish joint velocities directly, yielding smooth low-latency motion. A controller-switching utility toggles between the streaming velocity controller (teleoperation) and the trajectory controller (program execution, API commands).

#### 4.1.3. Data Collection and Dataset Pipeline

An Episode Recorder captures synchronized multimodal data during both teleoperation and autonomous operation: joint states at up to 50 Hz (HDF5), workspace and wrist-camera video (MP4), and program execution events (JSON), together with session metadata. Recorded episodes are converted into the LeRobot [43] dataset format with 7-dimensional state/action vectors (six joints plus gripper), enabling fine-tuning of foundation models such as $\pi_{0}$ [25] or OpenVLA [26] on domain-specific data.

### 4.2. Digital Twin

The framework includes a physics-based digital twin in NVIDIA Isaac Sim that replicates the physical workspace, including the UR5 with Robotiq 2F-85 gripper (Robotiq Inc., Lévis, Canada) and the objects declared in the task’s initial scene. The same .prog programs execute identically in simulation and on real hardware, allowing safe pre-flight of robot programs and decision policies.

Object meshes are reconstructed from reference images using SAM 3D [36] and imported as USD references with SDF-based collision approximations. Initial 6-DOF poses can be initialized from the perception pipeline given the camera-to-base extrinsic calibration, but mesh scale and pose still require manual adjustment to compensate for reconstruction error and collision-geometry simplification. Fully automating the adjustment is left to future work. For example, a closed-loop workflow or end-to-end trained model could be developed that replays a demonstration in the twin and optimizes object scale and placement until the simulation reproduces the reference video.

The Isaac Sim extension supports two operational modes: a ROS 2 Follower mode that mirrors /joint_states from the physical robot into the simulator at each physics step, and a VLA Control mode that executes actions from a Vision-Language-Action model through the simulator’s motion-commanded robot interface.

### 4.3. Evaluation Tasks

The AURA framework is task-agnostic: any collaborative scenario is added by authoring the configuration files of Section 3.7 and registering task-specific monitors where required. A more tightly specified task produces a behavior tree whose deterministic branches fire most decisions, reducing the LLM fallback rate. Robot skills are interchangeable at the catalog level: a .prog script, a cuRobo-planned trajectory, or a natural-language prompt dispatched to a learned VLA all share the same skill interface.

We instantiate the framework on three tasks spanning distinct operating points: composite hand layup (industrial, physical deployment, dense SOP), cuboid manipulation (reproducible, simulation-only, goal-rewritable at runtime), and tea preparation (domestic, speech-driven, mixed human/robot role assignment). Each is described in turn below.

#### 4.3.1. Task: Composite Hand Layup

Composite layup is a manufacturing process for fiber-reinforced polymer parts, widely used in aerospace and automotive industries [44]. It involves preparing the mold, mixing resin and hardener in precise ratios, positioning fiber plies, applying resin to each layer, debulking, and inspecting for defects. The task is well suited to proactive assistance: the human requires dexterity for ply placement while the robot can assist with tool provision, inspection, and resin application. We use a UR5 with a Robotiq 2F-85 gripper, with perception from a monocular workspace camera and a 360° wrist-mounted camera that keeps manipulated objects in view throughout each action.

**Figure 11:**
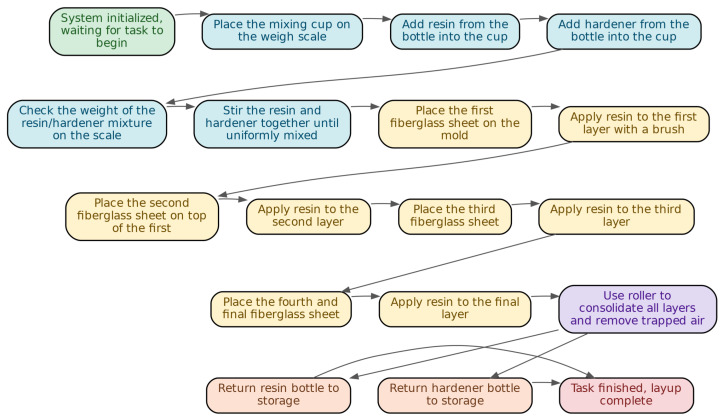
Task DAG for the hand layup procedure, visualized from its configuration file.

The task is encoded as a DAG of 18 nodes (Figure 11) over five phases: resin preparation (place cup on scale, add resin, add hardener, weigh, mix), layup (four cycles of place sheet and apply resin), consolidation, cleanup (return bottles to storage), and a terminal task_complete node.

The initial scene (initial_scene.json) declares two workspace regions (storage table and workplace) and the objects of interest: two bottles (resin, hardener), a mixing cup, brushes, a roller, a weigh scale, a mold, and fiberglass sheets. Object locations may be set explicitly or left as unknown for the perception monitor to resolve at runtime. The skill catalog defines 14 skills: six logistics programs (move/return each of resin, hardener, and roller between storage and workplace), a force-controlled roller consolidation skill, a table-cleaning skill, parameterized motion primitives, gripper open/close, and utility commands (speed, stop, wait). Each program declares preconditions on object locations and effects that update the SSG on completion. The state schema defines 15 variables tracked from video: current phase and action, counters for layers placed and resined, Boolean material flags (resin_added, hardener_added, mixture_mixed, consolidated), PPE compliance, predicted next action with confidence, robot status, and resin/hardener bottle locations. The cleanup phase exemplifies flexible role assignment: the human returns the hardener bottle while the robot returns the resin bottle.

**Figure 12:**
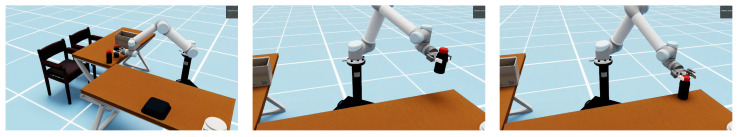
Digital twin in Isaac Sim executing a resin bottle transfer.

**Figure 13:**
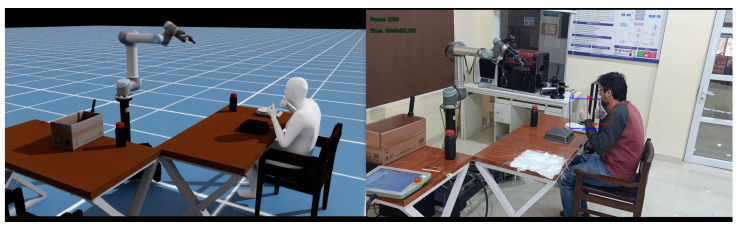
Side-by-side comparison of the Isaac Sim digital twin (**left**) and the real workspace (**right**).

The workspace is reconstructed as a digital twin following Section 4.2. Figure 12 shows a bottle transfer program executing in the twin; the same program runs on the physical UR5. Figure 13 compares the twin and real workspace, with the human represented as a body mesh recovered from video.  

To exercise the framework in a reproducible setting independent of physical hardware or participant recordings, we instantiate a lightweight cuboid manipulation task in Isaac Sim. The scene contains the same UR5 and Robotiq gripper, a fixed basket, and a small set of colored cuboids whose initial positions are randomized on reset. The scene combines simulator-bundled primitives with SAM 3D meshes released alongside the codebase, making the environment fully reproducible.

#### 4.3.2. Task: Cuboid Manipulation (Simulation)

The simulated robot shares the same control stack used on the real UR5, so the same task-level skill vocabulary, decision engine, and program format apply without modification. Live object poses are streamed out of the simulator over ROS 2 and consumed by the framework’s perception layer, replacing the vision-based perception monitors used in the physical experiments. The cuboid manipulation task is designed to be re-steered by editing the task configuration alone: by changing the declared goal, the set of available cuboids, or the target locations, the same underlying framework can be made to perform sorting, selective retrieval, stacking, or arbitrary user-specified rearrangements, without touching any framework code.

**Figure 14:**
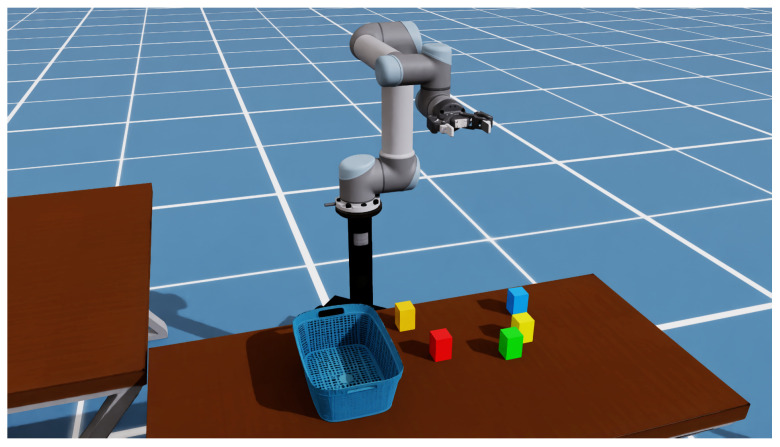
Cuboid manipulation scene in Isaac Sim.

Figure 14 shows the cuboid manipulation scene in Isaac Sim.

#### 4.3.3. Task: Tea Preparation

To illustrate that the framework generalizes beyond industrial manufacturing, a third configuration implements a domestic tea-preparation task. It is a long, loosely specified procedure whose only requirement is that an arbitrary user can hand the framework an egocentric recording of how they personally make tea, encode that preferred procedure as a DAG and state schema, and have the system follow along. We instantiate tea preparation across three recipes: the first two isolate the Intent Monitor and dispatch no robot actions, while the third drives the full end-to-end loop with the robot acting on the tracked state.

## 5. Results and Discussion

This section presents an empirical evaluation of our framework across the selected experimental tasks. We first validate the proposed Appropriateness Score against a selection of synthetic adversaries to confirm it ranks behavior sensibly. We then assess the accuracy of intention tracking on a procedural task. Finally, we evaluate the full system end to end: first on a domestic kettle tea-preparation task, then on an industrial hand-layup task.

### 5.1. Appropriateness Score Validation

**Figure 15:**
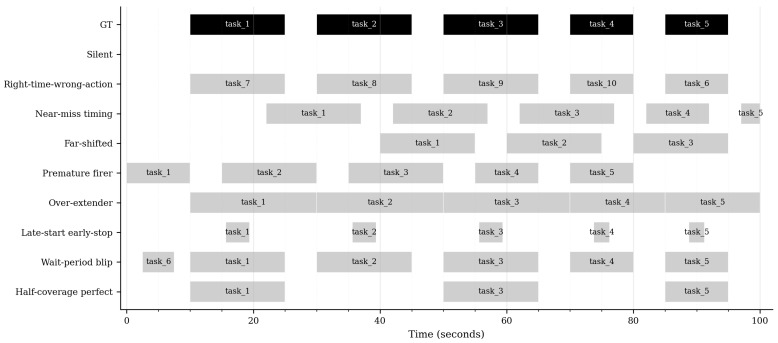
Synthetic ground truth versus each adversary’s prediction timeline.

Before applying the A-Score to the AURA framework, we verify that it ranks behavior sensibly relative to common alternatives. We construct a synthetic 100 s ground-truth track with five interventions separated by short WAIT gaps (so $T_{wait}=35$ s), and replay it against eleven hand-built adversaries that each target a specific failure mode (described in Table 2). The score is bounded in [0, 1] by construction: an oracle that copies G exactly attains 1, and a silent agent attains exactly the wait mass $w_{w}T_{wait}/Z$. Figure 15 stacks each adversary’s prediction track against the ground truth.

We compare the A-Score against four widely used alternatives: event-level F1 with a ±15 s start-time tolerance; the IoU averaged over matched pairs (mIoU); the action accuracy on matched pairs (mAcc); and a temporal action-agreement metric $P_{agree}$ that measures the fraction of task time at which the predicted active skill agrees with the ground-truth skill (or jointly equals WAIT). Table 2 pairs each adversary’s description with its score under all five metrics, including the task and wait decomposition of the A-Score.

The baseline metrics each fail on at least one adversary in a way the A-Score does not. Event-F1 is blind to action correctness: right-time-wrong-action and premature firer both earn F1=1.00 despite emitting wrong-skill or mistimed predictions. Matched-pair mIoU and mAcc are conditional on a successful match and therefore reward agents that simply do not predict: half-coverage perfect earns mIoU=mAcc=1.00 for the events it bothers to cover, hiding the two it drops. The A-Score reduces these to low scores by combining the per-event quality, the same-skill gating, and the wait-pollution term in one number. At the same time, the score is graded rather than binary: near-miss timing (0.24) and far-shifted (0.08) are correctly ordered by how far the predictions are from the ground truth, with no tolerance cliff between them; over-extender (0.76) is correctly registered as a mostly correct agent whose ends leak slightly into the wait region; and wait-period blip (0.97) is recorded as a near-oracle behavior with a small wait-period false positive.

The score also exposes its components for diagnostics. The task and wait contributions in Table 2 read directly: late-start early-stop keeps a clean wait term ($q_{wait}=1$) but its narrow predictions earn a middling task contribution (0.57); wait-period blip keeps a perfect task term but loses 0.03 on the wait term to a single 5-s false positive ($q_{wait}=0.86$); over-extender retains a high task contribution (0.65) but its 5-s end-padding leaks into the wait region, dragging $q_{wait}$ down to 0.46. This decomposition tells a grader which component of the schedule the agent failed to respect.

### 5.2. Intention Tracking on a User-Specified Procedure: Tea Preparation

The tea-preparation task is configured purely from a participant’s own first-person recording of how they prefer to make tea. The DAG (Section 4.3.3) encodes their idiosyncratic ordering, e.g., adding powdered milk to the cup early, asking about sugar preference only after pouring, and the framework’s only job is to follow along and keep an accurate estimate of what the human has done, what they are doing, and what they will do next. There is no robot intervention in this evaluation: it stresses the Intent Monitor end-to-end on long-horizon, loosely supervised egocentric video. We repeat the entire ablation on a second, independently sourced tea recipe: a boiled-milk preparation from a different participant in the public Ego-Exo4D dataset [45], reported alongside the powdered-tea task in the columns below.

We ablate two factors: (i) the VLM backbone, contrasting two locally hostable open-weight models (Qwen 3.5 0.8B and Qwen 3.5 4B) against the cloud-hosted Gemini 3.1 Flash Lite and Gemini 3.1 Pro; and (ii) the form of temporal memory threaded into the prompt by the Semantic Scene Graph, ranging from no previous state, to the monitor’s own self-reported previous state, to a ground-truth previous state injected from the keyframe annotations. The ground-truth condition is not a deployable configuration; it bounds how much of the residual error is attributable to the model’s degradation over time versus to the difficulty of the per-frame inference itself. We also report inference latency for each configuration.

**Figure 16:**
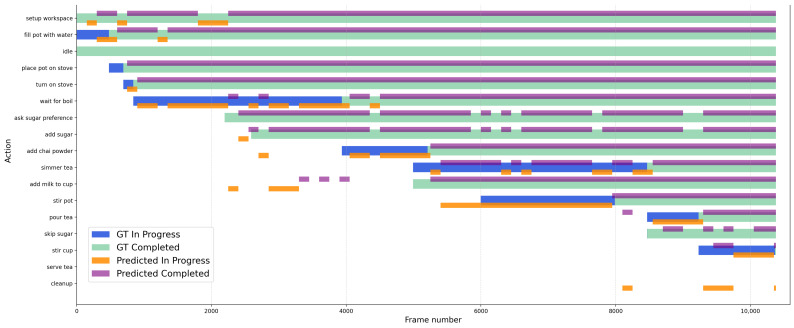
Intent-monitor output for tea preparation aligned against the ground-truth procedure (Gemini 3.1 Pro, ground-truth previous state).

A number of observations follow from Table 3. First, model scale dominates: the 0.8B local model collapses entirely (F1 =0.0983), reporting “Unknown” on most frames and hallucinating actions unrelated to the recording. The 4B variant recovers to roughly two-thirds of the cloud model’s performance. Premier reasoning-capable VLMs are presently required to track a user-specified procedure zero-shot from raw egocentric video. Second, the SSG-supplied memory matters even with a strong backbone: stripping the previous-state field from the prompt drops Gemini 3.1 Flash Lite from 0.669 to 0.6387 on Combined F1 and from 0.7214 to 0.6667 on per-frame state accuracy. Third, the gap between self-reported and ground-truth previous state (0.669→0.8339 Combined F1) shows that early errors compound: once the monitor mislabels an early step, it tends to remain anchored to the wrong branch of the DAG. Fourth, while Gemini 3.1 Pro slightly outperforms Flash Lite in accuracy (0.8440 vs. 0.8419), as visualized in Figure 16, its latency is nearly five times higher (17.1s vs. 3.5s mean), emphasizing the practicality of smaller multimodal backbones like Flash Lite for timely interactive assistance systems. The same broad pattern recurs on the independently sourced Ego-Exo4D milk-tea recording, produced by a different participant and following a structurally different recipe. The 0.8B model again collapses (Combined F1 0.018), and the 4B model recovers only partially (0.592), while both Gemini backbones track the procedure closely. Under ground-truth memory, Flash Lite reaches 0.872 Combined F1 and 0.9435 state accuracy, comparable to the far heavier Gemini 3.1 Pro (0.863/0.9247) at a fraction of its latency.

### 5.3. End-to-End Proactive Assistance on Tea Preparation

**Figure 17:**
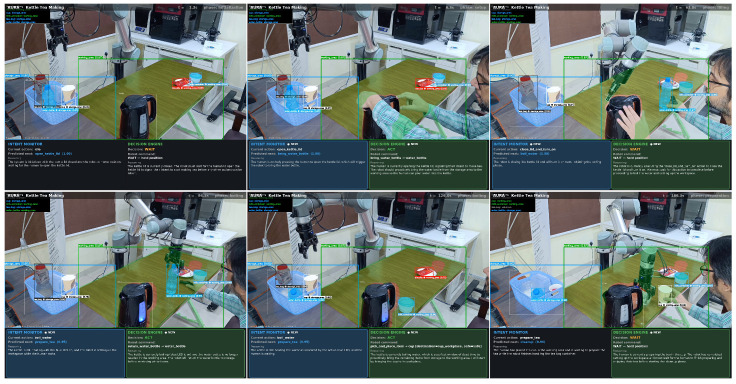
Representative frames from the kettle tea-preparation task, showing the outputs of the perception, intention, and decision modules.

We now evaluate full end-to-end proactive assistance, where the framework dispatches robot interventions in response to the tracked state. We begin with a domestic kettle-based tea-preparation task. The robot fetches and returns the items a person needs while they boil water and brew: it brings and later returns the water bottle, closes the lid and switches the kettle on, and stages the cup and the tea bag, returning the tea bag to storage afterwards. The ground-truth track contains six robot interventions, and the perception monitor is active for every run. This isolates the effect of the reasoning backbone. The ablations of perception and previous-state memory are studied on the hand-layup task in Section 5.4. Figure 17 shows representative frames from a run of the task.

The intervention repertoire is defined parametrically. In the skill catalog, the  two transfer skills pick_and_place_item(item, destination) and return_item_to_storage are single program templates that each instantiate an intervention for any object and region pair. The evaluation is consequently not restricted to a small, fixed set of interventions, and the same mechanism scales the assistance repertoire to an arbitrary number of staged objects.

**Figure 18:**
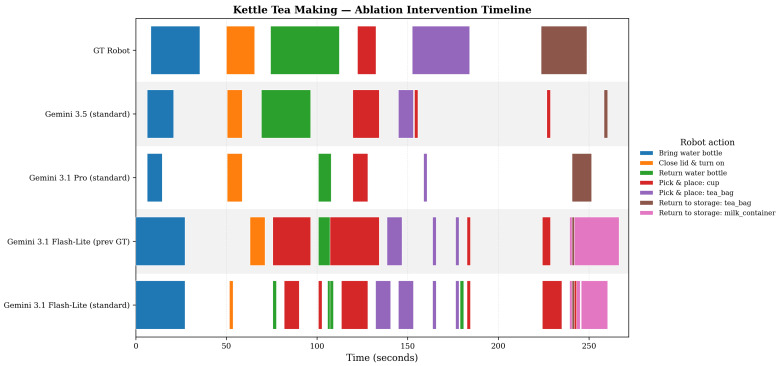
Intervention timelines for the kettle tea-preparation task across four reasoning backbones.

Table 4 reports the intent-monitor Combined F1, the end-to-end A-Score (Section 3.1.3), and the measured per-call intent-monitor latency for four reasoning backbones with perception fixed on; Figure 18 shows the corresponding intervention timelines against the ground truth. Two trends stand out. First, more capable backbones place interventions more cleanly. Within the lightweight class, the newer Gemini 3.5 Flash clearly outperforms the older Gemini 3.1 Flash-Lite on appropriateness (A-Score 0.623 vs. 0.447) at comparable intent accuracy, while the heavier Gemini 3.1 Pro is strongest overall (0.741 A-Score, 0.881 Combined F1). The lightweight model instead over-fires, emitting two to three times as many interventions as the ground truth, which appears as the dense thin bars in Figure 18. Second, injecting a ground-truth previous state raises the Flash-Lite Combined F1 to 0.850, increasing intention-tracking accuracy.

These results expose a trade-off between latency and appropriateness. The fastest backbone (Flash-Lite, about 4 s per call) is the least appropriate, and appropriateness rises together with model capability and, with it, latency (Gemini 3.5 at about 11 s and Gemini 3.1 Pro at about 15 s mean per call). The right balance is application-dependent: a robot’s perceived quality, legibility, and trustworthiness rest on the joint balance of timeliness, reliability, and predictability rather than on speed or accuracy alone [46]. We can also conclude that faster variants of the newest, most capable frontier models seem best placed to handle this challenging balance.

### 5.4. End-to-End Proactive Assistance on Hand Layup

The hand layup task exercises the complete framework: Intent Monitor, SAM 3-based perception monitor that tracks the resin and hardener bottles, deterministic precondition/effect arbitration over the skill catalog (robot_skills.json), and dispatch of UR5 .prog programs through the External Control API. We evaluate against four ground-truth robot interventions per session: move_resin_to_workplace, move_hardener_to_workplace, consolidate_with_roller_force, and return_resin_to_storage.

A deliberate choice in this evaluation is that the skill arbitration is made effectively deterministic. Each entry in robot_skills.json declares a trigger_after_steps list and a precondition block over SSG fields (e.g., resin_bottle.location = storage_area and resin_added = false for move_resin_to_workplace). Once those preconditions are satisfied in the SSG, the skill is dispatched without an LLM call. This deterministic behavior tree-style configuration allows us to attribute end-to-end errors to perception or intent rather than to non-reproducible LLM decisions, and it means the system’s output is directly explainable from the configuration: an operator wishing to retime an intervention edits the precondition rather than re-prompting the model. The flexibility of LLM fallback for ambiguous or undeclared situations remains available but is intentionally not exercised in the offline evaluation reported here.

We split the analysis in two layers. Table 5 reports the intent-monitor accuracy (Combined F1) on the hand layup video alongside the end-to-end Appropriateness Score (A-Score, Section 3.1.3) on the resulting robot interventions. It shows the performance across different ablations of the previous-state condition and whether the SAM 3 perception monitor’s bottle locations are visible to the intent prompt.

**Figure 19:**
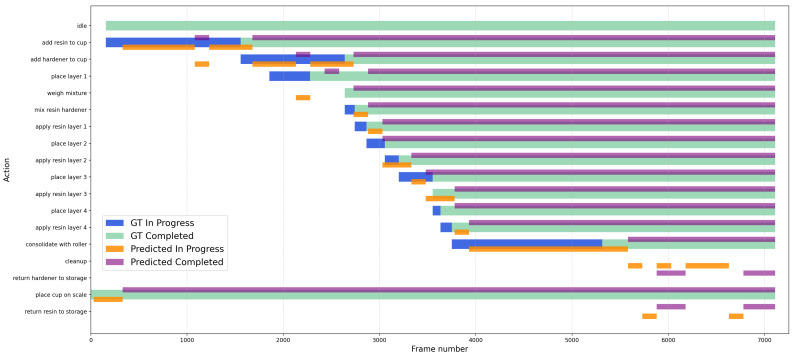
Intent-monitor output for the hand layup task aligned against the ground-truth procedure.

**Figure 20:**
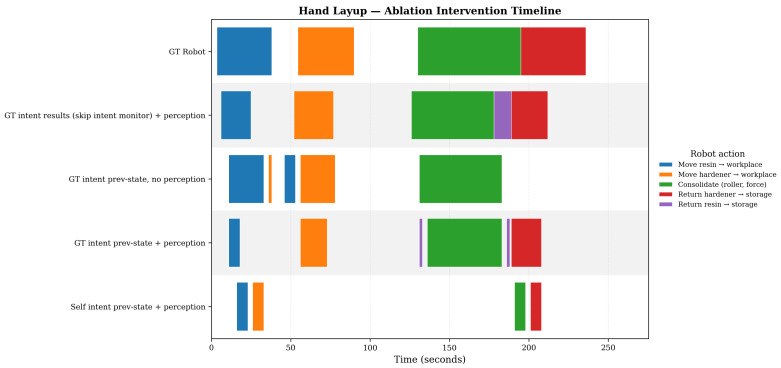
Intervention timeline comparison across four hand-layup ablations. The top track shows the ground-truth robot interventions; each subsequent track shows the predictions of one ablation.

We observe that the SAM 3 perception monitor improves both layers of the system. At the intent layer, exposing the tracked bottle locations to the prompt lifts Combined F1 from 0.8178 to 0.8623 in the ground-truth-state condition, because the monitor stops confusing which bottle is currently being handled (the performance of this intent tracking is presented in Figure 19). At the end-to-end layer, the performance degradation of removing perception is evident in Figure 20. The lightweight, real-time SAM 3 tracker is therefore not a cosmetic monitor but a precondition for correct arbitration.

In offline evaluation, the framework does not have access to a live robot status feed, so it cannot detect when a long-running intervention such as roller consolidation has actually finished. The completion of even visually obvious interventions (e.g., a handover, after which the arm still retracts to its home pose) cannot be read reliably from frames alone. In live mode, this signal is available through the External Control API. However, for offline evaluation, exposing the robot status from a recording that already reflects proactive assistance would leak the decision under test. Its effect on the reported scores is nonetheless limited, since in live operation a correctly issued command runs to completion without re-prompting, and offline this uncertainty enters the A-Score only as an end-time error Δe (Equation (4)). This highlights the challenge of evaluating and benchmarking collaborative robot actions in an offline setting.

### 5.5. Discussion

The experimental evaluations illustrate that SSG-mediated temporal memory is critical; without it, early errors compound during long-horizon procedures, substantially degrading state accuracy and intent prediction. Furthermore, continuous physical perception is not merely cosmetic. Grounded object state significantly improves both intent metrics and end-to-end Appropriateness Score (A-Score), proving it is a necessary precondition for reliable skill arbitration rather than a redundant signal alongside the VLM. Finally, deterministic precondition/effect arbitration ensures that execution errors remain directly attributable to perception or intent failures, rather than unpredictable LLM decisions. This makes the system’s behavior configuration-explainable, allowing operators to adjust intervention timing by editing explicit preconditions instead of re-prompting the model.

Furthermore, the human-in-the-loop teleoperation methodology effectively addresses a core challenge in proactive HRC research: establishing ground truth for inherently subjective assistance decisions. By capturing expert human judgment on intervention timing and action selection, this approach provides a robust basis for the quantitative and reproducible evaluation of robotic assistance algorithms without requiring repeated, variable human-subject studies.

## 6. Conclusions and Limitations

We presented AURA, a modular framework for proactive human–robot collaboration integrating a Semantic-Scene-Graph (SSG), a hybrid behavior tree/LLM decision engine, and zero-shot perception. Evaluated across three diverse tasks, results demonstrate that grounded perception and SSG-mediated temporal memory are critical for reliable proactive assistance. Furthermore, we defined the Appropriateness Score (A-Score) and a teleoperation-derived ground-truth methodology to benchmark intervention timing, utility, and correctness consistently.

The current framework relies on static configuration files, including the Directed Acyclic Graph (DAG) and other task definitions, to specify operating procedures. While users can easily modify these setups, the system cannot dynamically adapt them at runtime or autonomously learn from experience. This enforces linear execution and restricts responsiveness to unconstrained human behavior. A natural extension is an adaptive task-configuration module that closes this loop. A correction monitor could detect deviations from the SOP at runtime and propose corresponding updates to the task configuration. These updates could be based on observed deviations, the success or failure of past interventions, real-time input through voice, text, or a dashboard, or analysis of the system’s own inputs and outputs. This would let the framework refine its operating procedures during operation rather than relying solely on the authored setup. Furthermore, 6DOF tracking and perception extraction via models like SAM3 lack seamless extensibility, often requiring hardcoded heuristics or specific environmental adaptations, such as distinct markers or strategic object placement, to mitigate current model limitations. Additionally, ground-truth annotations in the human-in-the-loop teleoperation methodology are not easily scaled and the intervention timings depend on individual operator judgment.

A further limitation concerns the structure of the ground truth itself. The current A-Score formulation (Equation (3)) assumes each intervention is a single contiguous interval $I_{i}^{*}=\left[t_{s,i}^{*},t_{e,i}^{*}\right]$, which matches the hand-layup recordings used in this paper but is restrictive in tasks where assistance can be delivered through alternative multi-phase plans. A natural extension replaces each $I_{i}^{*}$ with a disjunctive temporal network [47], in which an intervention may be satisfied by any one of several alternative schedules. We leave this extension to future work.

AURA’s modular architecture allows seamless integration of additional monitors. This can include wearable sensors, such as heart rate sensors, electrodermal activity, or gaze tracking, to provide real-time indicators of an operator’s cognitive load, stress, or locus of attention. Similarly, the system can be extended to include environmental monitors or highly specialized task classifiers as required by specific manufacturing tasks. These new monitors can be added as modular components that feed into the shared Semantic Scene Graph, which will enhance the state representation available to the decision engine. This design allows the framework to be easily deployed to a wide range of collaborative scenarios, such as assembly, inspection, and packaging.

## Figures and Tables

**Table 2:** Synthetic adversaries and their scores on the validation timeline.

Adversary	What it Does	F1	mIoU	mAcc	$P_{\mathrm{agree}}$	Task	$q_{\mathrm{wait}}$	Wait	$A_{\mathrm{score}}$
Oracle	Copies GT exactly (sanity check).	1.00	1.00	1.00	1.00	0.76	1.00	0.24	**1.00**
Silent	Empty prediction track.	0.00	0.00	0.00	0.35	0.00	1.00	0.24	0.24
Right-time-wrong-action	GT intervals, action labels swapped from the catalog.	1.00	1.00	0.00	0.35	0.00	0.21	0.05	0.05
Near-miss timing	GT shifted by +12 s.	0.80	0.38	0.00	0.23	0.17	0.29	0.07	0.24
Far-shifted	GT shifted by +30 s.	0.75	0.36	0.00	0.20	0.00	0.35	0.08	0.08
Premature firer	GT shifted by −15 s.	1.00	0.00	1.00	0.15	0.09	0.23	0.06	0.14
Over-extender	Correct starts; every end padded by +5 s.	1.00	0.72	1.00	0.75	0.65	0.46	0.11	0.76
Late-start early-stop	Right action, only middle 25% of each GT fired.	1.00	0.25	1.00	0.51	0.57	1.00	0.24	0.81
Wait-period blip	Oracle plus one 5-s spurious event in the longest gap.	0.91	1.00	1.00	0.95	0.76	0.86	0.21	0.97
Half-coverage perfect	Every other GT event copied perfectly, the rest dropped.	0.75	1.00	1.00	0.75	0.46	1.00	0.24	0.70

**Table 3:** Intent-monitor ablation on tea preparation.

		Combined F1		State Acc.			
Model	Previous State (SSG Memory)	Powdered Tea	Milk Tea	Powdered Tea	Milk Tea	Mean Lat. (s)	P95 Lat. (s)
Qwen 3.5 0.8B	Self	0.0983	0.018	0.0606	0.0065	1.6	1.9
Qwen 3.5 4B	Self	0.543	0.592	0.5824	0.6028	5.7	6.0
Gemini 3.1 Flash Lite	None	0.6387	0.762	0.6667	0.8817	3.4	5.1
Gemini 3.1 Flash Lite	Self	0.669	0.799	0.7214	0.8871	4.1	6.5
Gemini 3.1 Flash Lite	Ground Truth	0.8339	**0.872**	0.8419	**0.9435**	3.5	5.2
Gemini 3.1 Pro	Ground Truth	**0.8673**	0.863	**0.8440**	0.9247	17.1	26.5

**Table 4:** End-to-end results on the kettle tea-preparation task.

Model	Combined F1	A-Score	Mean Lat. (s)	P95 Lat. (s)
Gemini 3.1 Flash-Lite (standard)	0.795	0.447	4.0	5.3
Gemini 3.1 Flash-Lite (prev GT)	0.850	0.424	4.0	5.3
Gemini 3.5 (standard)	0.814	0.623	10.7	15.3
Gemini 3.1 Pro (standard)	**0.881**	**0.741**	15.2	22.1

**Table 5:** Intent-monitor ablation and end-to-end Appropriateness Score (A-Score) on hand layup (Gemini 3.1 Flash Lite). Perception = SAM 3-based bottle tracking exposed to the intent prompt.

Previous State (SSG Memory)	Perception	Combined F1	A-Score
Ground Truth (skip Intent Monitor)	✓	—	0.828
Ground Truth	✓	**0.8623**	0.801
Ground Truth	×	0.8178	0.754
Self	✓	0.6476	0.556
Self	×	0.6716	—

## Data Availability

The open-source implementation of the AURA framework is available at https://usmanasad88.github.io/aura/ (accessed on 12 June 2026), and the robot control and simulation stack is available at https://github.com/usmanasad88/ur5-robotiq-ros2-control (accessed on 12 June 2026).
